# A Novel Microfluidic Device Integrated with Chitosan-Modified Capillaries for Rapid ZIKV Detection

**DOI:** 10.3390/mi11020186

**Published:** 2020-02-11

**Authors:** Xinchao Zhu, Jun Zhao, Anzhong Hu, Jingyu Pan, Guoqing Deng, Changyi Hua, Cancan Zhu, Yong Liu, Ke Yang, Ling Zhu

**Affiliations:** 1 Optoelectronic Application Technology Research Center, Institute of Applied Technology, Hefei Institutes of Physical Science, Chinese Academy of Science, Hefei 230031, Chinapanjy136@126.com (J.P.);; 2 University of Science and Technology of China, Hefei 230026, China; 3 Institute of Optical Engineering, Anhui Normal University, No. 1, Beijing East Road, Wuhu 241000, China

**Keywords:** in situ PCR, molecular diagnosis, microfluidic chip, POC device, ZIKV

## Abstract

The outbreak of Zika virus (ZIKV) has posed a great challenge to public health in recent years. To address the urgent need of ZIKV RNA assays, we integrate the microfluidic chip embedded with chitosan-modified silicon dioxide capillaries, smartphone-based detection unit to be a C^3^-system for the rapid extraction and detection of ZIKV RNA. The C^3^-system is characterized by: (1) four chitosan-modified silicon dioxide capillaries integrated in the microfluidic chip for target ZIKV RNA enrichment and “in situ PCR” (polymerase chain reaction) amplification; (2) smartphone-based point of care (POC) device consisting of a pneumatic subsystem for controlling the nucleic acid extraction processes in the microfluidic chip, a heating subsystem for sample lysis and PCR amplification, and an optical subsystem for signal acquisition. The entire detection processes including sample lysis, ZIKV RNA enrichment, and reverse-transcription polymerase chain reaction (RT-PCR) is achieved in the microfluidic chip. Moreover, PCR buffers can be directly loaded into the chitosan-modified silicon dioxide capillaries for “in situ PCR”, in which the captured ZIKV RNA is directly used for downstream PCR without any loss. ZIKV RNA extracted by the C^3^-system can be successfully recovered at very low concentrations of 50 transducing units (TU)/mL from crude human saliva. This means that our method of detecting viremia in patients infected with ZIKV is reliable.

## 1. Introduction

Zika virus (ZIKV) is a positive-sense single-stranded RNA virus, a member of the family flaviviridae, genus flavivirus [1]. According to the Centers for Disease Control and Prevention (CDC), 80% of cases of ZIKV-infected individuals are asymptomatic [2], while the symptoms in other cases are mild and nonspecific, including fever, rash, joint pain, conjunctivitis, myalgia, and headache [3]. It means that no symptom is unique for ZIKV infection, thus making differential diagnosis even more challenging. Though infected mosquitoes are the main disseminators for the transmission of ZIKV infections, the ZIKV gene can be detected in blood, saliva, urine or semen, indicating multiple potential routes of person-to-person transmission [4,5,6]. The asymptomatic or non-specific infection has many symptoms on ZIKV-infected individuals. ZIKV transmits through multiple ways and mostly spreads in resource-limiting areas urging an inexpensive, continent, and accurate method for ZIKV diagnose.

ZIKV infection can be diagnosed by testing the anti–ZIKV IgM antibodies of people; this is an instrument-free and low-cost approach, but its testing results can be misidentified to other homologous flaviviruses, such as dengue virus infection [7]. Reverse-transcription polymerase chain reaction (RT-PCR) is considered as a star standard for ZIKV diagnose according to the CDC [8]. However, conventional PCR progress is usually time-consuming, and requires well-trained experimenters, bulky clean bench, or commercial instruments for nucleic acids extraction, which are not suitable for filed deployment [9,10]. Point of care (POC) devices defined as instruments that can perform an analytical or diagnostic test near the site of resource-limited areas are urged to apply for epidemic disease control [11]. Integrated microfluidics technologies with POC devices, enable viral or bacterial pathogen diagnostics outside of laboratories. Furthermore, the combination of a microfluidic system with a high-efficiency DNA enrichment method significantly increases the pathogen detection sensitivity in a clinical application [12]. Unfortunately, the on-chip nucleic acid testing (NAT) usually requires elaborate designs and optimizations to achieve the balance between the reliability of the results and the complexity of the microfluidics chip with external equipment [13,14,15].

In order to reduce the redundant extraction steps on the microfluidic chip, some researchers proposed a novel sample preparation strategy, namely, in situ PCR. It is featured with sample preparation and nucleic acid amplification in the same chamber [16,17]. For in situ PCR, the PCR is directly carried out in the extraction chamber following nucleic acid purification without elution [15]. In this direction, a microfluidic chip that integrates aluminum oxide membrane-based nucleic acids isolation and PCR amplification in a single chamber was demonstrated. However, the hydrophobicity and fragility of the membrane make the microfluidic chip fabrication and operation complicated [17]. Further, several groups have used transverse porous membranes, such as glass fiber, filter paper, and cellulose membrane, for physical entanglement-based nucleic acid capturing and “in situ PCR” detection in practice [18,19,20]. In addition, several pH-sensitivity materials, such as chitosan or poly-L-lysine, were also used to modify the transverse porous membrane for nucleic acids separation based on the charge adsorption principles [21,22]. On the one hand, the nucleic acids are easily entangled by the membrane-based material that allows more DNA to be immobilized and reserved, even being washed multiple times. On the other hand, these methods are inappropriate for high-viscosity samples because the porous film or hydrophilic filter paper can clog up with sticky stuff. When the sticky stuff in the sample covers the membrane surface, the channel blockage and the flow resistance increase. Therefore, the samples need dilution or centrifugation before being tested, which inevitably increases the complexity of the operation. Furthermore, some commercial filter papers usually contain fluorescent molecules, which may affect the readout of the fluorescence signal [23].

Compared to porous film or hydrophilic filter, an alternative path is to directly use the modified inner surface of the extraction chambers for target nucleic acids absorbing. However, only few studies reported the use of modified microchannels or capillaries as the substrates for nucleic acids adsorption. For example, cationic poly diallyldimethylammonium chloride (PDDA) was coated on the glass capillaries based on the electrostatic self-assembly principle that permitted the DNA to be isolated from the solution [24,25]. In addition, thermoplastics with polar groups, such as poly methyl methacrylate (PMMA) and polystyrene (PS), were also used for DNA enrichment on their pristine surfaces. However, regardless of the PDDA-modified capillaries or the thermoplastic, both have no charge-switch ability. Although these materials can isolate the nucleic acids from the lysed samples during the extraction step, it is difficult to desorb fixed nucleic acids. In this way, the next PCR amplification process is definitely affected.

To solve the above problems, we developed a chitosan-modified capillary assist, microfluidic-based in situ PCR method for the rapid extraction and detection of ZIKV RNA in the study. Herein, chitosan-modified silicon dioxide capillaries were selected as the solid surface that could provide enough surface-area-to-volume ratio structure with many more selective adsorption sites. Furthermore, we integrated the microfluidic chip embedded with chitosan-modified silicon dioxide capillaries and smartphone-based detection unit to a C^3^-system for the rapid extraction and detection of ZIKV RNA in order to overcome the limitations of traditional nucleic acid extraction methods (e.g., labor-intensive, time-consuming, and difficulty of integration with the POC testing). Detailed compositions of the C^3^-system are shown in Section 2. Experimental results show that the C^3^-system is efficient and reliable in terms of isolating high-quality ZIKV RNA for in situ PCR analysis from biological samples. For ZIKV RNA obtained from lysed saliva samples, the detection of the C^3^-system is within the limit around 50 TU/mL, lower than the commercial method.

## 2. Materials and Methods

### 2.1. Materials and Chemicals

The silicon dioxide capillaries (with 0.9 mm inner diameter) were purchased from the Shengrui Chemical Co. Ltd. (Shanghai, China) and 200 μm thickness of elastic film were purchased from Bald Advanced Materials CO. Ltd. (Hangzhou, China). All chemicals were purchased from Sigma-Aldrich (St. Louis, MO, USA), including hydrochloric acid (HCl), sodium hydroxide (NaOH), chitosan, and 2-(N-morpholino)-ethanesulfonic acid (MES). All chemicals were analytical grade. The RT-PCR kits were purchased from TaKaRa Biotechnology (Dalian, China). All primers used in this research were synthesized by Sangon Biotech Co., Ltd. (Shanghai, China).

### 2.2. Sample Preparation

A kind of virus-like particle (VLP) that contained the special ZIKV RNA sequences was synthesized by FENGHUI Biotech Co. Ltd. (Hunan, China). The original concentration of VLPs was around 5 × 10^9^ TU/mL. The VLP was further spiked in human saliva to simulate a real ZIKV sample. In this study, we tested 0 (negative control), 5 × 10, 5 × 10^2^, 5 × 10^3^, 5 × 10^4^, and 5 × 10^5^ TU/mL ZIKV saliva samples. A total of 10 healthy adult donors provided saliva samples in our experiment, and all of the samples were mixed together to confirm homogeneity of the simulated matrix. All experiments were repeated three times.

### 2.3. Design and Fabrication of the Capillary Microfluidic Chip

**Figure 1:**
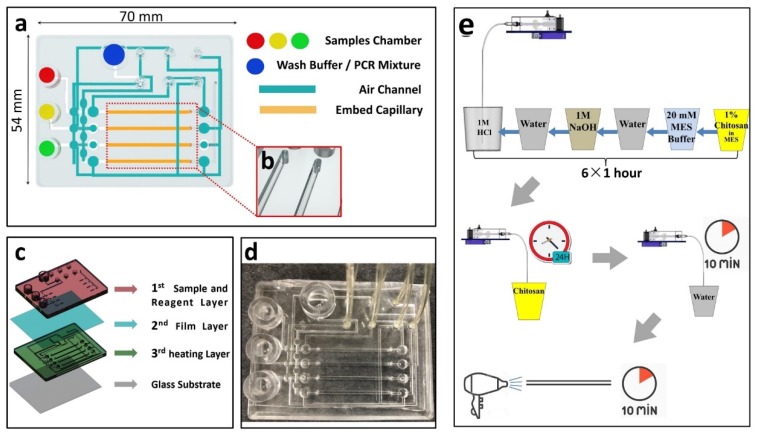
Microfluidic chip design. (**a**) Top view of the chip. Three sample chambers are arranged in parallel at the left end of the chip, containing different samples, such as serum, urine, or saliva solution, respectively. Polymerase chain reaction (PCR) mixture loaded in chamber at the top of the chip, and air source ports at the upper right corner of the chip. (**b**) Capillaries embed in the grooves. (**c**) A schematic hierarchy of the chip, from top to bottom is as follows: sample and reagent layer, film layer, heating layer, and glass substrate. (**d**) Photograph of microfluidic chip and tubes. (**e**) A schematic diagram of capillary modification progresses.

A polydimethylsiloxane (PDMS)-based microfluidic chip (70 mm length × 54 cm width × 9 mm height) was designed using the Solidworks software (Dassault Systemes, Vélizy-Villacoublay, France)(Figure 1). The microfluidic chip consists of four substrate layers, including the sample and reagent layer (top layer), film layer (middle layer), heating layer (third layer), and glass substrate (Figure 1c). Generally, the microfluidic chip detects three samples from an experiment with the three chitosan-modified capillaries within it (Figure 1a). The amplification chambers at a lower surface of the third layer consist of four chambers with width of 1.1 mm. Three chitosan-modified capillaries and one untreated capillary were embedded in the chambers (Figure 1b). In addition, this microfluidic chip integrates three samples’ chambers and one wash buffer/RT-PCR mix chamber. All of these samples and reagents flow into chitosan-modified capillaries, located in the third layer, through interconnected vertical tunnels.

### 2.4. The Progress of Capillary Modified by Chitosan Solution

The process of modification is shown in Figure 1e. The cleaning steps include: (1) swill the silicon dioxide capillaries by using hydrochloric acid solution with the concentration of 1 M for 1 h. (2) Use 1 M sodium hydroxide to swill the capillaries for 1 h. (3) Rinse the capillaries with deionized water for 1 h to confirm chaotic ions cleanout on the inner surface of glass capillaries. (4) Wash the silicon dioxide capillaries slowly by using 100 mM MES buffer (pH 5.0) for 1 h. The modification steps are as follows: (1) Dissolve cationic polyelectrolyte chitosan in 100 mM MES buffer (pH 5.0) to prepare the chitosan solution (1%, *w*/*v*). (2) Immerse the cleaned silicon dioxide capillaries into chitosan solutions for 24 h. (3) Take out the silicon dioxide capillaries and wash them for 10 min to clean out unthatched chitosan using ultrapure water. (4) Dry the silicon dioxide capillaries by air pressure for another 10 min to evaporate water adhered to on the surface of silicon dioxide capillaries. 

### 2.5. Custom-Made POC Device

**Figure 2:**
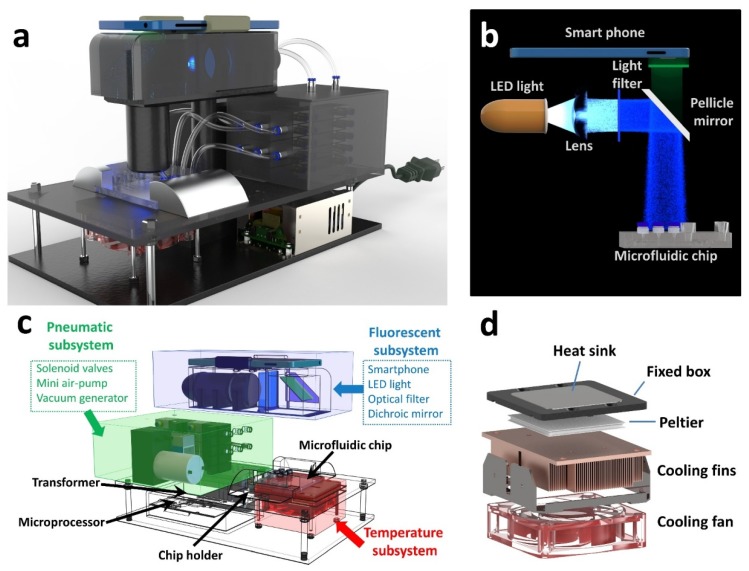
Point of care (POC) device design. (**a**) A stereogram of a POC device. (**b**) Schematic diagram of optical subsystem, which consists of a smartphone, a light emitting diode (LED) light, a lens, two light filters, and a pellicle mirror. After the LED light is focused by the lens and passes through the filter, a bundle of parallel exciting light is reflected by the pellicle mirror, and the returned emission light is captured by smartphone. (**c**) A perspective drawing of the POC device. Each subsystem is filled with an individual color—blue, green, and red—to correspond to fluorescent detection subsystem, pneumatic subsystem, and temperature control system, respectively. (**d**) Schematic diagram of temperature control subsystem, which consists of a heat sink, a fixed box, a Peltier element, cooling fins, and a cooling fan.

Figure 2 illustrates a schematic of the custom-made equipment and each component inside the equipment before and after the assembly process. A drawing of the POC device (with dimensions of 31 × 22 × 26 cm) is shown in Figure 2a, including a smartphone, a fluorescent detection subsystem with the smartphone fixed to the device case for fluorescence collection, a temperature control subsystem for maintaining temperature conditions for nucleic acids extraction and amplification, a pneumatic subsystem consisting of solenoid valves and an air pump for controlling the pneumatic micro valves opening and closing in the microfluidic chip, and a microprocessor to coordinate the various subsystems and communicate with the smartphone via Bluetooth. The distribution of each component is shown in Figure 2c.

The fluorescent detection subsystem was used for exciting and collecting the amplification signal in capillaries, as shown in Figure 2b. Briefly, a diverging blue light produced by a light emitting diode (LED, 470 nm, CREE, Durham, NC, USA) was collimated by lens. The centralized light was first filtered by an optical filter (470 nm, Bd-optic. Ltd., Beijing, China), reflected at a 90° by a dichroic mirror (520 nm, Bd-optic. Ltd., Beijing, China), and irradiated on the capillaries embedded in the chip [26]. Fluorescence excitation light which was excited by the blue light inside the parallel capillaries was passed through the dichroic mirror towards the smartphone camera. The light was captured by the smartphone at each extension stage during PCR amplification.

The pneumatic subsystem is composed of ten solenoid valves (3-way, 6 mm, poppet valve, MAC Valves Inc., Wixom, MI, USA), a mini air-pump (Shenzhen Huacheng Science and Technology Co., Ltd., Shenzhen, China), and a vacuum generator (SMC Co., Ltd., Tokyo, Japan). All of the microvalve groups in the microfluidic chip can be opened or closed independently with different switching combinations of solenoid valve arrays [27]. Injection, removal, and operations in the microfluidic chip were manipulated through a negative pressure generated by an additional manual syringe. A detailed operation process is described in the Section 3.

The temperature control subsystem, as we reported previously [28,29], was used to maintain temperature conditions for sample lysis and PCR progress, as shown in Figure 2d. It consists of a heat sink wrapped in a fixed box, a Peltier element (Shenzhen Yangming Hongye Technology Co., Ltd., Shenzhen, China), cooling fins, and a cooling fan. The Peltier element, which is composed of two different conductors, has the ability to heat and cool with the same module. The heat sink made of double-sided polished silver was fixed above the Peltier element to stabilize the temperature. The cooling fins and cooling fans were located under the Peltier element to increase the cooling rate. A thermistor (M222, Heraeus Co., Ltd., Hanau, Germany) was located at the lower end of the heat sink to monitor the temperature through changes in current. Precise temperature control was achieved through the proportion integral differential (PID) algorithm inside the microprocessor. The temperature control subsystem was controlled wirelessly by a smartphone application under the Bluetooth module.

### 2.6. Adsorption Kinetics of Nucleic Acid in Capillaries Modified by Chitosan

Yeast RNA (Sangon Biotech Co. Ltd., Shanghai, China) was used to evaluate the adsorption equilibrium time. For the adsorption equilibrium experiment at pH = 5.0, the initial concentration of yeast RNA (Sangon Biotech Co. Ltd., Shanghai, China) was set as 80 ng/μL. Firstly, yeast RNA medium was introduced into 11 batches of chitosan-modified capillaries, 8 cm in length. The solution in part of the capillaries was recovered during a range of time (5, 10, 20, 30, 45, 60, 90, 120, 180, 240, and 300 s), and residual RNA samples were measured by a spectrophotometer (Biophotometer Plus, Eppendorf, Hamburg, Germany). The quality of adsorbed RNA was calculated by the difference between the initial concentration of the RNA and the residual one.

An isotherm model experiment was designed to further understand the adsorption characteristic. Similar with the experiment mentioned above, other batch of modified capillaries was divided into eight groups, and each group had twenty capillaries with an average length of 8 cm. The adsorption time was designed for 300 s. The volume of each group was 1 mL, and the experiment was carried out at 25 °C. The experiment data were analyzed by a non-linear regression method in ORIGIN 2019 software.

### 2.7. Preparation of in Situ PCR on the Chip

Before in situ PCR, the microfluidic chip was fixed at the heat sink. A layer of thermally conductive silicone grease was evenly coated between the microfluidic chip and the heat sink to increase the heat transfer effect. The samples and reagents were pre-injected to the storage chambers in the microfluidic chip. For ZIKV RNA extraction, 20 µL of sample and 20 µL of lysis buffer (10% protease K, 0.7 M NaCl, 0.1% Hexadecyl trimethyl ammonium Bromide (CTAB) and MES at pH 5.0) were added into the sample storage chamber in the microfluidic chip. Since the ZIKV samples in our experiment were replaced with VLPs as the ‘simulated samples’, which were different from the real samples, the real ZIKV may be partially parasitic in oral exfoliated epithelial cells, rather than free in the saliva matrix. However, the CTAB in the lysis buffer can rupture cell membranes, causing ZIKV in the cells to be released into the saliva matrix. That means our lysis condition is also suitable for real ZIKV samples.

In addition, 360 µL of deionized water (wash buffer) was added into the reagent storage chamber. After finishing RNA extraction, 200 µL of PCR mixture containing 160 µL tris(hydroxymethyl)aminomethane hydrochloride (Tris-HCl) solution (0.01 M, pH 8.5), 10 µL of reverse transcriptase, 20 µL of deoxy-ribonucleoside triphosphate (dNTP), 10 µL of the primers, 2 unit/µL Taq polymerase, and 1 × TB green dye were added into reagent storage chamber for the next RT-PCR reaction. As a positive control, the same samples were subjected to RNA extraction by using a commercial RNA magnetic bead extraction kit (Qiangen Co., Ltd., Dusseldorf, Germany) and then the extracted nucleic acids were mixed with PCR reagent and amplification on the C^3^-system.

### 2.8. Fluorescence Signal Acquired and Data Analysis

The fluorescence intensity in the microfluidic chip was captured by the complementary metal oxide semiconductor (CMOS) sensor on a smartphone at each end of the extending process. The difference in fluorescence intensity between the experimental group and the control group is sufficient to make qualitative judgment on the samples. However, for quantitative analysis as well as real-time PCR, an additional personal computer was needed. The pictures captured by the smartphone were imported to a private computer for further data analysis. The average fluorescence intensity obtained from 3–15 cycles of each amplification chamber was set as the baseline, and the standard deviation (SD) of each sample was calculated. The curves of fluorescence intensity were fitted by the fast Fourier transform method, and cycle threshold (Ct) values of each sample were defined as the sum of baseline and 3 times the SD. The data were processed and analyzed in ORIGIN 2019 software (OriginLab Inc., Northampton, Massachusetts, USA) and MATLAB 2017a (MathWorks Inc., Natick, MA, USA).

## 3. Results

### 3.1. Nucleic Acids Extraction Strategy on the Capillary

**Figure 3:**
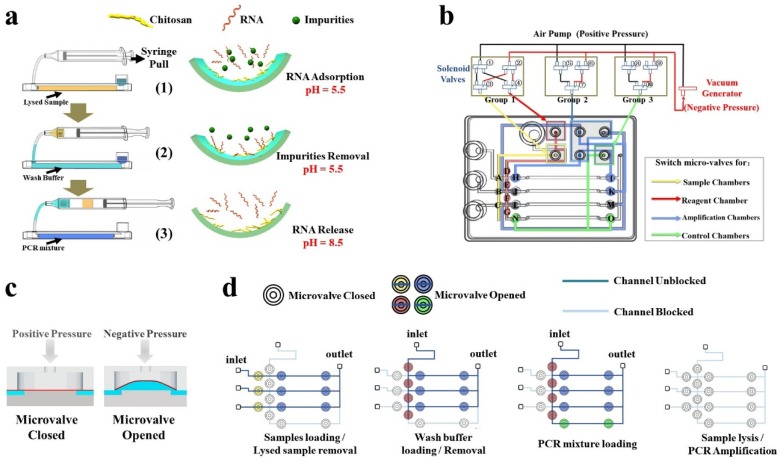
Working principle of the chip. (**a**) Illustration of individual sample process by a single capillary chamber based on charge-switch ability of chitosan, corresponding to lysed sample, wash buffer and PCR mixture were loaded into a capillary chamber by a unidirectionally moving piston. The target nucleic acids were adsorbed in the acidic solution (pH 5.5) and desorbed into PCR mixture in alkaline solution (pH 8.5). (**b**) The distribution diagram of liquid flow channels (uncolored channels), air flow channels (colored channels), microvalves (lettered) on the chip and corresponding solenoid valves (numbered). (**c**) The figure of the microvalves. The deformation of the membrane in the microvalve was affected by the air pressure to control the blocking and unblocking of the corresponding channel. (**d**) The schematic diagram of the four working states of the microvalve on the microfluidic chip, respectively are: samples loading and lysed samples removal, wash buffer loading or removal, PCR mixture loading, sample lysis or PCR amplification.

The illustration of the individual sample process by a single capillary chamber is shown in Figure 3a. A syringe was used to transfer the sample or reagent from the storage chamber to the capillary by piston pull motion. Under negative pressure generated by the syringe, the liquids were loaded into a chitosan-modified capillary from the storage chamber, or sucked into the syringe cavity from the capillary, as the cavity was considered a waste chamber. Based on the charge-switch ability of chitosan, the progress of nucleic acids purification was easy. The process was as follows: Firstly, a lysed sample (with yellow color, pH 5.5) was loaded into the chamber, and nucleic acids were adsorbed by the modified capillary through electrostatic interaction. Secondly, the lysed sample was sucked into the syringe cavity, while the wash buffer (with sky blue color, pH 5.5) was introduced into the capillary for surface cleaning by washing away the free impurities. Since the piston was moved unidirectionally throughout the operation, PCR inhibitors in the lysed sample drawn into the syringe no longer contaminated the capillary. Thirdly, the PCR mixture (with blue color, pH 8.5) was introduced into the capillary. Then the negatively charged chitosan returned to a neutrally charged state and the adsorbed nucleic acids were released into the solution. Since the two kinds of liquid were not directly contacted, the mixture was not diluted by residual wash buffer to effect subsequent operations.

Nucleic acid extraction strategy on the chip is one of the most critical procedures of sample preparation. It directly determines complexity of the step and the amount of time consumed. The main methods of nucleic acid extraction, like conventional solid-phase extraction (SPE) and magnetic beads (MBs), require multiple washing and elution steps removing PCR inhibitors such as organic solvent residual and hard-to-simplify structures [30,31]. Chitosan, a natural cationic polysaccharide, is an ideal material for RNA purification. Here, the chitosan-modified capillary-based SPE method has distinct advantages compared with conventional methods. First, it is simple and easy to fabricate, and the plunger of the syringe performs all operations by only moving unidirectionally. Second, based on the charge-switched characteristic of chitosan, the purity of extracted RNA is guaranteed. As the pKa of chitosan is around 6.3, it is positively charged and enables the enrichment of anionic RNA in acidic solution (pH 5.5) from a complex substance due to the electrostatic adsorption. When the PCR mixture (pH 8.5) is introduced into the capillary, the chitosan tends to be electrically neutral and RNA releases into the solution [21]. Third, single-stranded RNA is fragile and easily degraded by RNAses that are widely present in the environment, thus the process of RNA extraction often needs to be performed in a clean bench. Chitosan molecules condense efficiently with nucleic acids forming polyplexes and preventing its degradation by RNAses [32]. In this way, our device could deploy in some resource-limited area.

In order to achieve multi-channel molecular diagnosis and avoid cross infection between samples, we designed a four-channel microfluidic chip with multi-microvalves for blocking different chambers. The whole C^3^-system is illustrated in Figure 3b with the detailed status of each solenoid valve (numbered), pressure inlet, liquid flow channels (uncolored channels), air flow channels (colored channels), and microvalves (lettered) distribution indicated. There are six air source interfaces in the upper right corner of the chip, which were divided into four groups, corresponding to control microvalves (A, B, C) for blocking sample reagent chambers through the yellow air flow channel, microvalves (D, E, F, G) for blocking wash buffer/PCR mixture chambers through the red air flow channel, microvalves (H, I, J, K, L, M) for blocking sample amplification chambers through the blue air flow channel, and microvalves (N, O) for blocking negative control chambers through the green air flow channel, respectively. A mini air-pump provides positive pressure and the pressure was divided into two paths: one path to seal microvalves on the chip, and the other path to connect to a vacuum generator to provide negative pressure for open microvalves on the chip. Deformation of the membrane in the microvalve was affected by the air pressure to control the opening and closing of the corresponding channel, as shown in Figure 3c. The air pressure generated by the air pump to seal the microvalves is higher than that of 200 kPa. During PCR thermal cycling, when the temperature increases to 95 °C, the increased pressure generated by vapor is about 84.5 kPa, which is sufficient to be sealed by pressure from the air pump.

The solenoid valves numbered 1–4 were used to switch the microvalves which control sample storage chambers or reagent storage chambers, while those numbered 5–7 and 8–10 were used to control microvalves for amplification chambers and negative control chambers, respectively. The sheet of opened/closed microvalves and energized solenoid valves in different procedures on the chip is shown in Table 1 and the schematic diagram is shown in Figure 3d, corresponding to four kinds of working states: (1) Samples are loaded and lysed samples removed; at this working state, the microvalves that control the sample chambers and amplification chambers were opened. Under the negative pressure formed by the syringe, the samples enter the amplification chamber and are lysed, and then transferred into the chamber of the syringe. (2) During wash buffer loading or removal, the wash buffer can be introduced into the amplification chambers for impurities removal. (3) During PCR mixture loading, the PCR reagent can be loaded into four of the amplification chambers. (4) Under the state of sample lysis or PCR amplification, the amplification chambers are sealed. By controlling the application on the smartphone, the switching of each group of solenoid valves is realized, so that the microvalves on the microfluidic chip are in different working states.

### 3.2. Nucleic Acids Purification, Amplification, and Qualitative Detection on the C^3^-System

**Figure 4:**
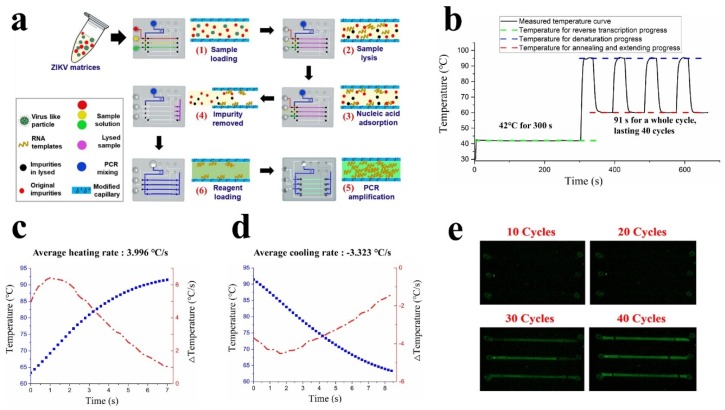
(**a**) Schematic diagram of the Zika virus (ZIKV) detection process on the microfluidic chip, including six steps: (**1**) Samples (RNA solution or ZIKV matrices mixing with lysate) loading into three capillaries. (**2**) Sample lysis in the capillaries. (**3**) Nucleic acids adsorption. After lysis process, the liquid remained in the capillaries for one minute, and target nucleic acids were expected to be adsorbed by capillaries. (**4**) Any impure liquid was removed from the port of the chip after RNA adsorption process. (**5**) RT-PCR reagent loading. (**6**) Executed RT-PCR process on the chip. (**b**) The curves of measured temperature during PCR process. The product was 42 °C for 5 min for reverse transcription, 95 °C for 15 s for denaturation, 60 °C for 40 s for primer annealing and extending, lasting 40 cycles. The time cost of an individual PCR cycle was around 91 s. (**c**) The figure of heating rate of the device. The blue curve represents the measured temperature during the heating process, and the red curve represents the heating rate. (**d**) The figure of the cooling rate of the device. (**e**) The images captured by the smartphone at different cycles during PCR amplification.

The whole process of ZIKV diagnostic is done using the C^3^-system; one-step in situ RT-PCR is schematically shown in Figure 4a and the label illustration is inherited from Figure 3b. The microvalves were all controlled to be closed before the experiment assays. Firstly, virus samples mixing with lysis buffer and wash buffer were pre-loaded into the corresponding chambers, and the microvalves were switched to the working state for ‘sample loading’ through an application on the smartphone. Then the samples were slowly introduced into capillaries with a unidirectionally-moving piston. Secondly, when the capillary chambers were filled with the sample, the microvalves at the two ports of the capillaries were controlled to close, and samples were heated for lysis process. In order to fully lyse the sample, inactivate protease K, and allow the chitosan-coated capillaries to capture as much RNA as possible, it is necessary to incubate at 65 °C for 5 min and at 90 °C for 2 min. Thirdly, the microvalves were switched to the working state for ‘wash buffer loading’. Then the wash buffer chamber and the sample capillary chamber were unblocked, while sample chambers were blocked; then the wash buffer (pH 5.5) was introduced into the capillaries for waste removal. As the negative-charged RNA can be adsorbed by chitosan in an acidic solution environment, the target nucleic acids kept binding at the surface of the modified-capillary until wash buffer was transferred. At the same time, the residual impurities like ions and lysed protein fragments were further washed out. Fourthly, the microvalves were controlled to switch to the working state for ‘PCR mixture loading’ and the negative capillary chamber was unblocked, then the pre-loaded PCR mixture was introduced into all of four amplification chambers. Finally, while the amplification chambers was immersed by the PCR mixture, the microvalves were controlled to seal all of the chambers. The process of nucleic acids purification can be finished within 25 min. Compared to tedious and time-consuming (60 min or more) methods based on commercial kits, our method for nucleic acids purification is much easier and more flexible.

For PCR amplification, the product was set at 42 °C for 5 min for reverse transcription, 95 °C for 15 s for denaturation, 60 °C for 40 s for primer annealing and extending, lasting 40 cycles, as shown in Figure 4b. Since the time cost of an individual PCR cycle is around 91 s, the entire PCR process (including step of the reverse transcription) could be finished in 65 min. The heating and cooling rate of our C^3^-system was 3.996 °C and −3.323 °C on average during the amplification process, as shown in Figure 4c,d. Since the temperature sensor was located on the heat sink rather than inside the microfluidic chip, the time in denaturation and extending stages was deliberately extended in order to confirm the temperature of the amplification chambers consistent with the expected temperature. However, the PCR amplification efficacy was nearly 100% (see Section 3.6), indicating that the temperature control performance of our device achieves effective PCR amplification.

The fluorescence excitation light generated by fluorochrome could be captured by the smartphone camera at each end of the extending field. In order to avoid the interference of external ambient light, the device needs to be blocked by an opaque black cloth during the amplification process. As the number of cycles of PCR amplification increases, the positive samples gradually become brighter and are observed directly, as shown in Figure 4e. Limited by the input power and cooling efficiency of the device, the rate of rising and falling in temperature of our C^3^-system is slightly weaker than that in commercial instruments. By combining with microfluidic technology and chitosan-based nucleic acid purification strategy, our portable device realized qualitative detection of nucleic acids in 90 min.

### 3.3. Characterization of Chitosan Modified Capillary and Confirmation of Adsorption Performance

**Figure 5:**
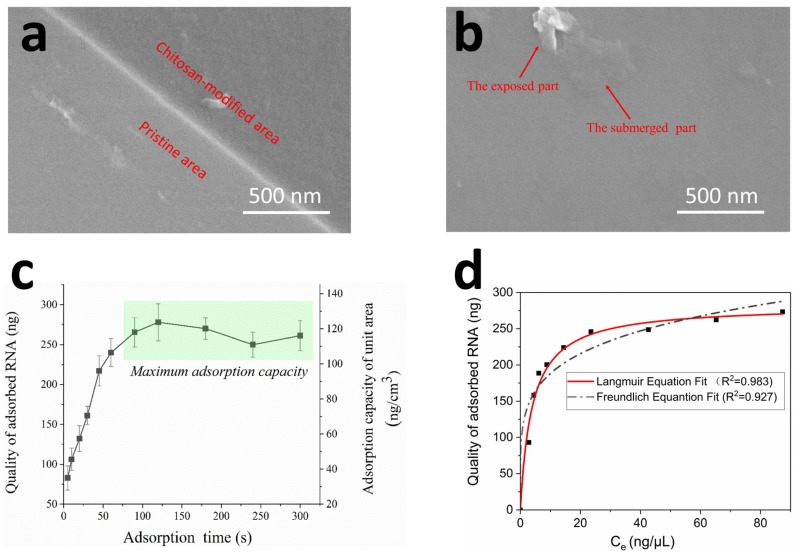
(**a**) The boundary between the modified area and unmodified area on the capillary surface; (**b**) Inner surface of chitosan-modified capillary, a granular substance lay on the surface of the modified capillary and the lower half of the particle appears submerged in a layer of gel (the submerged part), with the top half of the particle exposed outside (the exposed part), indicating that the form of chitosan modified on the capillary was a successive gel-like film; (**c**) Capacity of RNA separation at different times; (**d**) The Freundlich and Langmuir fits for experimental adsorption data of RNA.

Before the experiments, we observed the morphology of chitosan covering the surface of the capillaries with a field emission scanning electron microscope (FESEM). As seen in Figure 5a, the pristine inner surface of the capillary (lower left side in the picture) was clearly smooth and light-grayish. In addition, the modified inner surface of the capillary (upper right area in the picture) was changed to uneven and charcoal grey after chitosan treatment, indicating the successful modification of chitosan on the surface of the capillary. Furthermore, as shown in Figure 5b, a granular substance lay on the surface of the modified capillaries and the lower half of the particle appears submerged in a layer of gel, which means the form of chitosan modified on the capillary was a successive gel-like film.

Next, we evaluated the effect of adsorption time on the quality of the RNA that was enriched by the modified capillaries, as is shown in Figure 5c. The quality of the RNA separated by the capillaries increased rapidly from 5 to 90 s. The adsorbing capacity did not further increase when the incubation time exceeded 90 s, indicating that the nucleic acid adsorption reached equilibrium between chitosan-based capillaries and the RNA. We consider that additional adsorption steps are unnecessary, as the sample lysis and RNA enrichment processes were performed simultaneously. For a single chitosan-modified capillary, the upper limitation of RNA adsorption capacity was approximately 240–280 ng, and unit adsorption capacity was in the range of 105–130 ng/cm^2^, indicating that our modified capillaries have adequate nucleic acid capacity ability.

We then analyzed the distribution of RNA on adsorbents through an isotherm model experiment. As a method of electrostatic adsorption, the adsorption of negatively charged nucleic acids by positively charged chitosan in acidic solution, might fit the Langmuir model or Freundlich model, which are two conventional models to describe isotherm equations [33,34,35]. The equations of Langmuir and Freundlich isotherm models are given by the following:

(1) $$Langmuir:\;Q_{eq}=\frac{Q_{m}K_{L}C_{e}}{1+K_{L}C_{e}}$$

(2) Freundlich: Qeq=KFCe1n

where $Q_{eq}$ is the adsorbed substance by modified capillary and $C_{e}$ is the equilibrium concentration of substance in the remaining solution, $Q_{m}$ is the maximum adsorption capacity of the capillary, $K_{L}$ is the Langmuir constant, $K_{F}$ is the adsorption capacity of adsorbent (Freundlich model), and 1/*n* is the adsorption intensity [36]. The adsorption isotherm curves of RNA on the capillaries are shown in Figure 5d and the equation parameters are shown in Table 2. High R^2^ is derived by fitting experimental data into the Langmuir isotherm model (R^2^ > 0.982), as compared with the Freundlich isotherm model (R^2^ > 0.927), suggesting that the Langmuir isotherm model rather than the Freundlich isotherm model generates a satisfactory fit to the experimental data [37]. The Langmuir model considers that interaction between adsorbed species (RNA) and adsorbent is due to the homogeneous distribution of adsorbed molecules on active sites of the modified capillary [36]. Thus, the RNA is easier for desorption or amplification in subsequent processes.

The range of ZIKV-specific RNA fragments in our experiment is between 200–300 bp, which has same order of magnitude as the yeast RNA fragment. Nevertheless, the real ZIKV fragment is much longer than the above (with a genome of 10.2 kb) [1]. The adsorbed nucleic acids create an exclusion zone to other incoming nucleic acids based on their physical sizes and electric potentials. Once the electrostatic exclusion zones start to overlap, further adsorption of more nucleic acids is disfavored [38]. Thus, the long fragment of ZIKV may have a weakened adsorption capacity compared with the short fragment at high concentration. However, since the concentration in our next amplification experiment is far from the saturation concentration, the effects of the electrostatic exclusion zone are ignored.

### 3.4. Optimization of Wash Conditions

**Figure 6:**
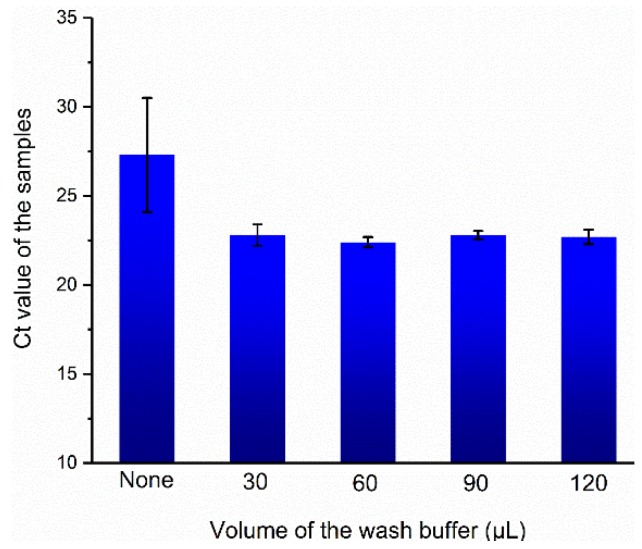
Results of relationship between washing volume and cycle threshold (Ct) values of amplification.

Prior to testing the in situ PCR in our platform, we optimized the wash buffer volume to remove PCR inhibitors instead of adsorbing nucleic acids during wash progress. The volume of continuously flowing wash buffer was 0, 30, 60, 90, and 120 µL for a single chamber respectively, and the result is shown in Figure 6. When the wash buffer volume of the solution are 30, 60, 90, and 120 µL, the corresponding Ct values for subsequent amplification are 22.8 ± 0.6, 22.4 ± 0.2, 22.8 ± 0.2, and 22.7 ± 0.4, respectively. However, the Ct value of amplification without wash step was much higher than that of others (27.3 ± 3.2). As hydroxyl-rich chitosan-modified capillaries are hydrophilic, the lysate solutions were residual in the form of droplets even after lysate was transferred away, thus PCR inhibitors in lysate composition such as CTAB or NaCl may retain. We demonstrate that even small volumes of wash buffer allow residual inhibitors to be removed during the wash process, meanwhile the wash buffer does not result in nucleic acid loss.

### 3.5. Real-Time PCR on C^3^-System

**Figure 7:**
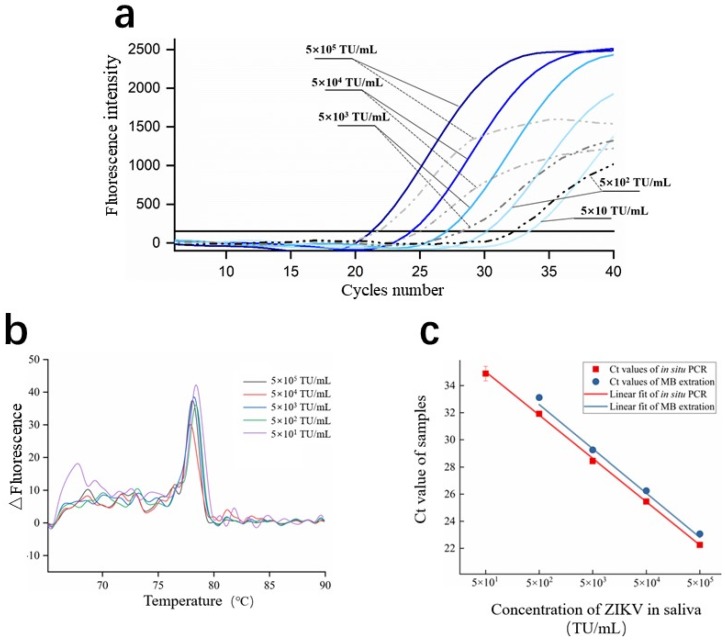
Results of ZIKV RNA sequence amplification in the C^3^-system. (**a**) Amplification curves of ZIKV mixed with saliva on the chip, including method of the capillaries adsorption (solid blue curve) and the kit extraction (dash grey curve). (**b**) Melting curve of sample with a range of concentration. (**c**) Regression curve of concentration of ZIKV samples.

Figure 7a depicts the fitted fluorescence emission intensity as a function of cycle number during the amplification process of the RT-PCR on C^3^-system, including the method of capillary adsorption (solid blue curve) and kit extraction (dash grey curve). The fluorescence emission intensity of the negative control remained below the baseline during whole process of amplification, indicating negligible amplicon formation and primer-dimers. The Ct values of capillaries adsorption with concentrations of 5 × 10, 5 × 10^2^, 5 × 10^3^, 5 × 10^4^, and 5 × 10^5^ TU/mL were 34.8 ± 0.5, 31.9 ± 0.2, 28.4 ± 0.2, 25.5 ± 0.2, and 22.3 ± 0.1, while the Ct values of the RNA extracted by Qiangen® RNA magnetic bead extraction kit were none, 33.1 ± 0.2, 29.3 ± 0.1, 26.3 ± 0.2, and 23.1 ± 0.2, respectively. The samples extracted by commercial kit with concentration of 5 × 10 TU/mL were not detected, indicating that our novel method showed better performance for nucleic acids enrichment.

To confirm that the fluorescence signals specifically came from the PCR amplification of the target ZIKV template, we compared the melting curve of several samples from 5 × 10 to 5 × 10^5^ TU/mL. As is shown in Figure 7b, all of the melting curves showed the same Tm value, indicating that specific amplification. No signal appeared in the negative control group. For 5 × 10 TU/mL, a relatively weak melting peak appeared between 65 and 70 °C, which means that the primer-dimer appeared simultaneously with the amplification of the target sequence. However, although the melting peak of the primer-dimer was relatively weak compared with the normal peak, no influence was exerted for the judgment of positive samples. We confirmed that the detection limit of our method was 50 TU/mL or lower, which was approximately 2.5 × 10^3^ genome equivalents (ge)/mL (calibration by RNA standard solution, data was not shown). As typical viremia of a patient infected with ZIKV ranges from 4.2 × 10^5^ ge/mL to 5.2 × 10^6^ ge/mL in saliva samples [33], our method is more than sufficient to detect viremia in patients infected with ZIKV.

### 3.6. Amplification Efficiency of In Situ PCR

PCR amplification efficiency is one of the most important indicators that directly determines the concentration of the sample detected. The theoretical PCR amplification efficiency is within 90%–110%, but this could be affected by factors such as inhibitors in samples, primers adsorption during amplification, or temperature accuracy of the device. To calculate PCR amplification efficiency of our method, the Ct values of the concentration gradients of both extraction methods were fitted to construct calibration curves, as shown in Figure 7c. The calibration curves are shown in the following equation:

(3) Y=aX+b

where *X* is the concentration of sample before amplification, *Y* is the Ct value of the sample after amplification, and *a* is the slope of the calibration curve, which corresponds to the amplification efficiency of the nucleic acids extracted by the capillaries or MBs. The parameters of both calibration curves are shown in Table 3. The amplification efficiency of RNA extracted by the capillaries was 98.3%, which was almost identical to the efficiency of purified RNA extracted by MBs (98.1%), indicating that in our chitosan-modified capillaries adsorption and amplification method, there were no inhibition effects in PCR amplification process, such as inhibitor residue or primers adsorption. What is more, the feature that the quality of chitosan-modified capillaries adsorbed RNA has a linearity with the original sample concentration is proven. It means that quantitative PCR could be performed with this method for ZIKV or other virus diagnoses with an additional private computer.

Some researchers indicated that PCR inhibition could appear in the method of in situ PCR; for example, Ian et al. [39] captured plasmid DNA by chitosan microparticles and the direct PCR amplification efficiency was 67.6%, which was much slower than theoretical efficiency. This phenomenon may be due to the presence of highly dense chitosan adhering on particles or cellulose; some part of the surface could not be converted entirely to electrical neutrality, which caused adsorption effect beside the template with adsorbents, such as protein, PCR inhibitor, primer, or enzyme in nucleic acid extraction or amplification process. Though Gan [21] demonstrated that DNA can physically entangled with the chitosan modified fiber at pH higher than 8.5, restricted nucleic acid fragments were limited to contact with primersf or enzymes, thus effecting PCR amplification efficiency. Nanayakkara et al. [40] put the DNA-adsorbed chitosan-coated microparticles directly into the PCR reagents for amplification. However, both the fluorescence peak intensity and the Ct value of their method lagged behind the result of DNA amplification in solution. They assumed that the difference was caused by inaccessibility of some nucleic acids, either for the agminated particles in the chamber which prevented most of the particle surface area from being accessed or for some of the targeted nucleic acid which were bound too tightly within the beads. The in situ amplification within our C^3^-system whose target RNA adsorbed by capillaries fitted the Langmuir model, adsorbed target RNA homogeneous distribution on the surface of the capillaries, and the targeted RNA sequence could touch with primer or enzyme accessibly, thus exhibiting near 100% amplification efficiency.

## 4. Conclusions

In conclusion, we developed a novel pathogen diagnosis facility named C^3^-system, based on the concept of in situ PCR for easy, fast, and quantitative ZIKV RNA assays. Several outstanding characteristics make our system a candidate for POC devices: (1) Through the features of the chitosan with pH responsive ‘on and off’ switches, nucleic acids are adsorbed/desorbed in lysate/PCR mixture environment respectively, enabling rapid isolation and purification of nucleic acids in the extraction step; (2) miniaturized equipment, takes full advantage of a smartphone, making the device portable, and achieves qualitative detection of nucleic acids in saliva samples; (3) as adsorbed nucleic acids were homogeneously distributed on the surface of the adsorbing medium, the RNA could accessibly contact the primer or polymerase, thus no PCR inhibition occurred during amplification. As a proof of concept, crude saliva samples mixed with VLPs contained sequences of ZIKV whose RNA was isolated in the microfluidic chip. It was suitable for RT-PCR detection with high purity and integrity and the results were comparable to that of a commercial nucleic acid extraction kit but with a simpler procedure. This portable, easy-to-use, and rapid molecule diagnostic platform provides great potential for pathogen diagnostics, which can be used in resource-limited environments.

## Figures and Tables

**Table 1:** Opened/closed microvalves and energized solenoid valves in different procedures.

Procedure on Chip	Opened Microvalves	Closed Microvalves	Energized Solenoid Valves
**Samples loading/lysed samples removal**	A B C H I J K L M	D E F G N O	1 4 6 8 10
**Wash buffer loading/removal**	D E F G H I J K L M	A B C N O	2 3 6 8 10
**PCR mixture loading**	D E F G H I J K L M N O P	A B C	2 3 6 9
**Sample lysis/PCR amplification**	D E F G	A B C H I J K L M N O	2 3 5 7 8 10

**Table 2:** Isotherm parameters according to Freundlich and Langmuir fit for RNA adsorption.

Parameter of the Langmuir Fit			Parameter of the Freundlich Fit		
$Q_{m}$	$K_{L}$	*R* ^2^	$K_{F}$	*N*	*R* ^2^
282.4	0.255	0.982	119.7	5.094	0.927

**Table 3:** Parameters of calibration curves of in situ PCR and magnetic beads (MBs) extraction.

Parameter of the Linear Fit of In Situ PCR				Parameter of the Linear Fit of MBs Extraction			
Slope	Intercept	R^2^	Efficiency	Slope	Intercept	R^2^	Efficiency
−3.192	8.47	0.998	98.3%	−3.189	10.17	0.994	98.1%
